# Cultivable Microbial Diversity Associated With Cellular Phones

**DOI:** 10.3389/fmicb.2018.01229

**Published:** 2018-06-07

**Authors:** Rashmi Kurli, Diptaraj Chaudhari, Aabeejjeet N. Pansare, Mitesh Khairnar, Yogesh S. Shouche, Praveen Rahi

**Affiliations:** National Centre for Microbial Resource, National Centre for Cell Science, Pune, India

**Keywords:** mobile phones, microbial contamination, MALDI-TOF mass spectrometry, de-replication, high-throughput identifications, objectionable microorganisms

## Abstract

A substantial majority of global population owns cellular phones independently to demographic factors like age, economic status, and educational attainment. In this study, we investigated the diversity of microorganisms associated with cellular phones of 27 individuals using cultivation-based methods. Cellular phones were sampled using cotton swabs and a total of 554 isolates representing different morphotypes were obtained on four growth media. Matrix-assisted laser desorption ionization time of flight (MALDI-TOF) mass spectrometry could generate protein profiles for 527 isolates and species-level identification was obtained for 415 isolates. A dendrogram was constructed based on the protein profiles of the remaining isolates, to group 112 isolates under 39 different proteotypes. The representative strains of each group were selected for 16S rRNA gene and ITS region sequencing based identification. *Staphylococcus*, *Bacillus*, *Micrococcus*, and *Pseudomonas* were the most frequently encountered bacteria, and *Candida*, *Aspergillus*, *Aureobasidium*, and *Cryptococcus* were in case of fungi. At species-level the prevalence of *Micrococcus luteus*, *Staphylococcus hominis*, *Staphylococcus epidermidis*, *Staphylococcus arlettae*, *Bacillus subtilis*, and *Candida parapsilosis* was observed, most of these species are commensal microorganisms of human skin. UPGMA dendrogram and PCoA biplot generated based on the microbial communities associated with all cellular phones exhibited build-up of specific communities on cellular phones and the prevalence of objectionable microorganisms in some of the cellular phones can be attributed to the poor hygiene and sanitary practices. The study also revealed the impact of MALDI-TOF MS spectral quality on the identification results. Overall MALDI-TOF appears a powerful tool for routine microbial identification and de-replication of microorganisms. Quality filtering of MALDI-TOF MS spectrum, development of better sample processing methods and enriching the spectral database will improve the role of MALDI-TOF MS in microbial identifications.

## Introduction

Since the dawn of human civilization man has been developing new tools, a cellular phone is one such, portable electronic device. They are now affordable, easy to use, comfortable and equipped with almost every latest feature we desire such as a calculator, internet, social media, games, camera, and many more. With recent advances in the source of information and social media apps, cellular phones have become an indispensable accessory in social and professional life (Pal et al., 2015). In less than 20 years, cellular phones have gone from being rare and expensive pieces of equipment used primarily by business elite to pervasive low-cost personal item (Ekrakene and Igeleke, 2007). The electromagnetic radiation emitted from phones and base stations has a threat to lives because the electromagnetic radiation has been reported to alter the electric activity of the brain causing sleeplessness, headache, malaise, memory retentiveness, and low sperm quality (World health organization, 2014). Cellular phones are now being used almost everywhere; whether it be the dining table, the kitchen, a restaurant, the gym, or even the toilet resulting in continues exposure of cellular phones to different types of microorganisms. Being an electronic gadget, cellular phones are seldom cleaned. All these factors and the heat generated by cellular phones, has been considered as the major contributors to the harboring of microbes on the device at alarming levels (Al-Ghamdi et al., 2011; Brady et al., 2011). Many microbes are resistant to desiccation and can persist on phone surfaces for weeks and daily contact with the face, ears, and hands may pose a direct health risk of getting an infection from cellular phones infested with microbes (Cosgrove et al., 2007; Chaibenjawong and Foster, 2011).

Colonization of bacteria has been observed on cellular phones resulting in a possible spread of nosocomial infections due to frequent contact (Ulger et al., 2009; Beckstrom et al., 2013). Majority of the microbial communities recovered from the cellular phones are similar to their owner’s hands or other body parts (Ulger et al., 2009; Brady et al., 2011; Beckstrom et al., 2013; Kiedrowski et al., 2013; Meadow et al., 2014). Based on the potential to spread the infections cellular phones have been considered as “Trojan horses” for pathogenic infection (Walia et al., 2014). Application of sound personal hygiene could check the microbial load on cellular phones and thus prevent the possible disease transmission through cellular phones. It is obvious that the majority of the microorganisms on cellular phone surfaces represent a transient population, which might get away by just slight rubbing of the surface of cellular phones (Rahi et al., 2018). However, it is also well known that several microorganisms can colonize non-living and inert substrates by forming biofilms. In addition to this, surfaces of cellular phones also get greasy and sticky by handling them with unclean hands. These sticky and greasy surfaces might allow colonization of diverse microorganisms. At the same time, the environment in which the individual lives and work may also influence the microbial diversity of cellular phones. Until now, most of the studies on cellular phone microorganisms were performed on the health-care workers targeting the survey of the pathogenic microorganism (Ulger et al., 2009; Brady et al., 2011; Beckstrom et al., 2013). In this study, we tried to generate basic information on the diversity of microorganisms colonizing cellular phones of individuals working under different environments other than clinical setups.

Quick and reliable identification of microorganisms was always a challenge for microbiologists. With the advent of MALDI-TOF MS based microbial identification, it is now possible to characterize the microorganisms in few minutes. Recently, MALDI-TOF MS has become the technique of choice for the fast and low-cost identification of a wide range of microbes including bacteria, archaea, and fungi especially in clinical set-ups (Rahi et al., 2016). In addition to the speed and low-cost, MALDI-TOF MS based biotyping also allows high-throughput microbial identification and is the technique of choice in microbial culturomics (Lagier et al., 2016; Rahi et al., 2016). The major objective of this study was to accurately identify the microorganisms colonizing cellular phones up to species-level, to generate more conclusive results on the presence of objectionable microorganisms. To achieve this objective, we used MALDI-TOF MS technique, which is fast, accurate, and low cost. Since we were using this technique for microbial identifications, we pose another objective to address the challenges in acquiring good quality MALDI-TOF MS spectrum and made some recommendation to improve the MALDI-TOF MS based identification of microorganisms.

## Materials and Methods

### Sampling

Twenty-seven volunteer participants working in different departments, including laboratories, offices, canteen, and security at the National Centre for Cell Science, Pune were approached for sample collection and requested to sign the prior informed consent. The cellular phones were collected in separate sterile bags and transported to the laboratory. Sterilized swabs moistened with phosphate buffer saline were used to swab the microbes from the screens of cellular phones. The samples were further processed for isolation of microorganisms in four different media.

### Isolation and Preservation of Microorganisms

Two general media were used for the isolation of microorganisms including Trypticase Soy Agar (TSA) for bacteria and Sabouraud Dextrose Agar (SDA) for fungus. To isolate opportunistic enteric pathogens MacConkey’s agar and to culture fastidious pathogenic bacteria, yeasts, and molds Brain Heart Infusion Agar (BHIA) (HiMedia, Mumbai, India) were used. An aliquot of 100 μl swab suspension was spread over agar plates of all four media and plates were incubated at 30°C. Based on the morphological differences (shape, size, elevation, surface, edges, color, structure, the degree of growth, and nature) the colonies were isolated to acquire pure cultures by streaking them on the respective media. The spread plates were observed for 10 days to isolate the slow growers.

### MALDI-TOF MS Based Characterization

A smear of actively grown bacteria (single colony) was made as a thin film directly onto the spot on a MALDI target plate. The bacterial smear was overlaid with 1 μl saturated solution of alpha-cyano-4-hydroxycinnamic acid (HCCA) matrix prepared in 50% acetonitrile and 2.5% trifluoroacetic acid and allows to dry at room temperature. The samples were analyzed using Autoflex speed system (Bruker Daltonik GmbH, Germany). Mass spectra were acquired in a linear positive ion extraction mode at a laser frequency of 1000 Hz within a mass range from 2,000 to 20,000 Da. The ion source 1 voltage was 19.5 kV, ion source 2 voltage was maintained at 18.2 kV, lens voltage at 7 kV, and the extraction delay time was 240 ns. The spectra were calibrated externally using the standard calibration mixture (*Escherichia coli* extracts including the additional proteins RNase A and myoglobin, Bruker Daltonics). The MALDI Biotyper software 3.0 (Bruker Daltonik) was used to identify the isolates and to visualize the mass spectra.

A simple extraction protocol was employed to analyze the bacterial and fungal sample for which no spectra could be generated by direct analysis. Loopful of actively grown cultures were harvested for bacteria and yeast, while for mycelial fungal cultures biomass was harvested from liquid cultures. The microbial biomass was mixed thoroughly with ethanol (70% v/v) and the suspended cells were centrifuged at 10,000 rpm for 2 min. The supernatant was carefully discarded without disturbing the pellet and dried pellet was suspended in formic acid (70% v/v) by vigorous mixing followed by the addition of acetonitrile. The mixture was centrifuged again at 10,000 rpm to separate the pellet and 1 μl of clear supernatant was placed on a MALDI target. The extracted samples were analyzed in the Autoflex speed system by following the similar procedure used for the direct samples. Species-level identity was considered for the isolates with biotyper score value <2.0, while the MALDI-TOF MS analysis for the isolates with score value ranging from 1.7 to 1.99 was repeated to achieve the higher score values. A dendrogram was constructed using MALDI Biotyper software 3.0 (Bruker Daltonik GmbH, Germany) to group the isolates, for which Biotyper database search score value was <2.0.

### Sequencing of 16S rRNA Gene and ITS-Region

High-quality genomic DNA was extracted from the strains following the protocol of ZR Fungal/Bacterial DNA Micro Prep^TM^ Kit (Zymo Research). The 16S rRNA gene sequence was amplified using universal primers (27f: 5′-AGAGTTTGATCCTGGCTCAG-3′ and 1492r: 5′-TACGGCTACCTTGTTACGACTT-3′) according to the methods described by Gulati et al. (2008). The amplification of ITS 1, 5.8 ribosomal RNA gene and ITS 2 was achieved using the primers ITS 1: 5′-TCC GTA GGT GAA CCT GCGG-3′ and ITS 4: 5′-GCT GCG TTC ATC GAT GC-3′ following Rahi et al. (2009). The amplified products were directly sequenced using the ABI PRISM Big Dye Terminator v3.1 Cycle Sequencing kit on a 3730xl Genetic Analyzer (Applied BioSystems). The sequence data obtained was assembled and analyzed using DNA sequence assembling software Lasergene SeqMan Pro (DNASTAR Inc.). The similarity search of newly generated 16S rRNA gene sequences was performed against the type strains of prokaryotic species with validly published names available in the EzBioCloud’s database (Yoon et al., 2017) and ITS region against the unified system for the DNA based fungal species linked to the classification ver. 7.2 in the Unite database (Kõljalg et al., 2013).

### Data Analysis

To compare the total bacterial and fungal taxa recorded from cellular phones belonging to the individuals from four different study groups (i.e., canteen staff, laboratory staff, office staff, and security staff) the statistical analysis tool STAMP v2 (Parks et al., 2014) was used. A dendrogram was constructed using average neighbour (UPGMA) method and Principal Coordinate Analysis (PCoA) was carried out for the presence-absence of the bacterial and fungal taxa. The shared and unique microbial taxa the study groups were represented by a Venn diagram constructed by using online tool Venny ver.2.0 (Oliveros, 2007).

## Results

### Isolation and Identification of Microorganisms Associated With Cellular Phones

Overall, a total of 554 microbial isolates were selected based on their distinct morphological features from 27 cellular phones for identification in this study. Among these isolates, 515 were with smooth colonies and 39 were with mycelial colonies. MALDI-TOF MS spectra were generated for all isolates with smooth colonies and only for 12 isolates with mycelial colonies. The comparison of MALDI-TOF MS spectra of isolates to the biotyper database exhibited species-level identification based on more than 2.0 score value for 292 isolates in the first round of identification. The MALDI-TOF MS based identification was repeated for the remaining isolates and species level identification was achieved 123 isolates taking the total count of species-level identified isolates to 415.

The dendrogram generated based on the MALDI-TOF MS profiles of 112 isolates including 100 smooth colony isolates and 12 isolates with mycelial colonies, for which no reliable species-level identity could achieve, were clustered under 39 major groups. Sixty-six isolates including 39 representative isolates of MALDI-TOF MS dendrogram clusters, and 27 isolates of mycelial fungi were selected for sequencing based identification. 16S rRNA gene sequences were generated for 34 isolates, while fungal ITS region sequences were generated for 32 isolates. Sequencing based identification of isolates placed them under 15 different genera and 30 different species (**Supplementary File S1**). Twenty-seven mycelial isolates (for which no MALDI-TOF MS spectrum generated) belongs to 24 different species of the 16 fungal genera (**Supplementary File S1**).

**FIGURE 1:**
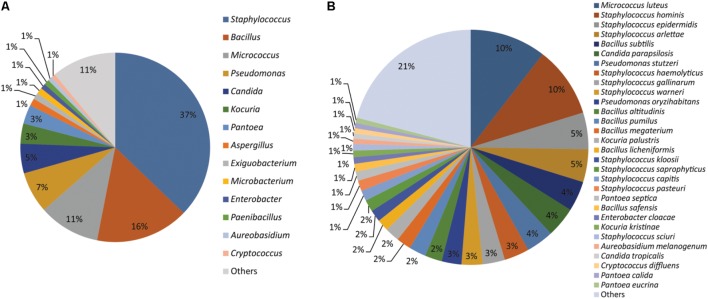
Microorganisms isolated from the cellular phones **(A)** genus-level identification, and **(B)** species-level identification.

Together the results of MALDI-TOF MS and sequencing based identification of the 554 microbial isolates represented 107 different microbial species. Of which, 76 species belong to 31 bacterial genera and 31 species belongs to 20 fungal genera (**Supplementary File S2**). *Staphylococcus* (37%), *Bacillus* (16%), *Micrococcus* (11%), *Pseudomonas* (07%), *Candida* (05%), *Kocuria* (03%), *Pantoea* (03%), *Aspergillus* (01%), *Exiguobacterium* (01%), *Microbacterium* (01%), *Enterobacter* (01%), *Paenibacillus* (01%), *Aureobasidium* (01%), and *Cryptococcus* (01%) were the dominant genera isolated from the cellular phones (**Figure 1A**). Nine species including, *Micrococcus luteus* (10%), *Staphylococcus hominis* (10%), *Staphylococcus epidermidis* (05%), *Staphylococcus arlettae* (05%), *Bacillus subtilis* (04%), *Candida parapsilosis* (04%), *Pseudomonas stutzeri* (04%), *Staphylococcus gallinarum* (03%), *Staphylococcus haemolyticus* (03%), and *Staphylococcus warneri* (03%), represented more than 50% of the total microbial diversity (**Figure 1B**).

### Microbial Diversity Across the Cellular Phones

**FIGURE 2:**
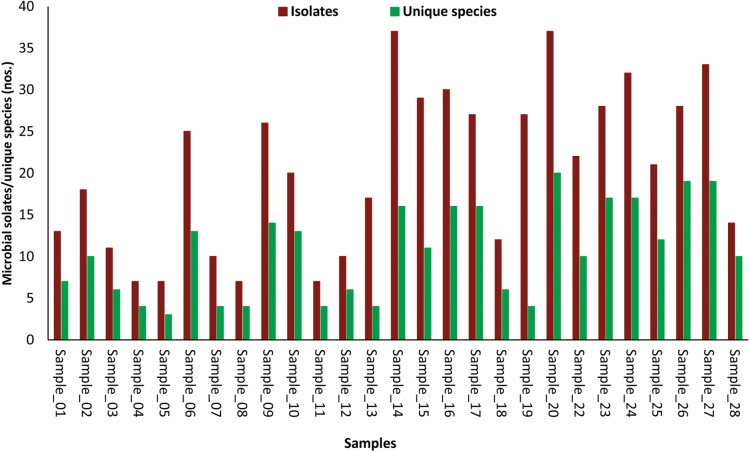
Bar-diagram depicting the number of morphotypes isolated and number of microbial species identified from individual samples of cellular phones.

The number of morphotypes picked was significantly high to that of identified microbial species indicated by low (1.1E-09) *p*-values of the paired *t*-test (**Figure 2**). Eight cellular phones were categorized as highly diverse, as they harbor more than 15 unique microbial species. Another set of eight cellular phones were moderately diverse and represented 10 to 14 unique microbial species each, and the remaining 11 cellular phones were least diverse as the number of unique microbial species was ranging between three to seven. The highest number of unique microbial species (20) were identified for cellular phone sample 20, while the cellular phone sample 05 showed the lowest number of unique microbial species (03) (**Figure 2**).

**FIGURE 3:**
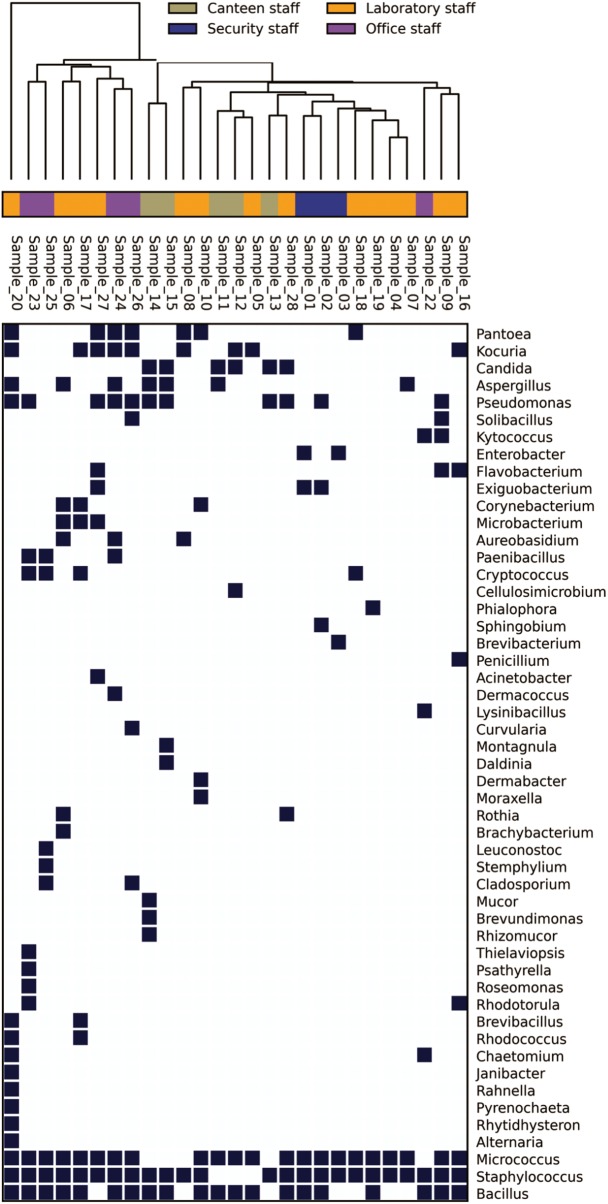
Genus-level distribution of microbial communities isolated from the samples collected of cellular phones.

**FIGURE 4:**
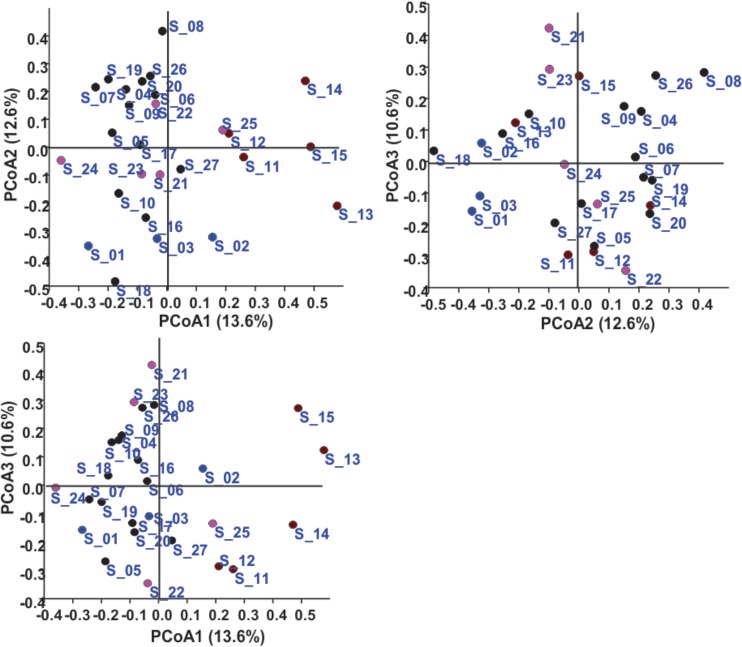
Principal coordinate analysis based on the microbial communities isolated from the samples collected of cellular phones. Circles in different colors represent sample collected from the cellular phones of individuals working in laboratories (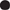), in offices (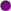), in canteen (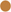), and in security gates (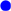).

**FIGURE 5:**
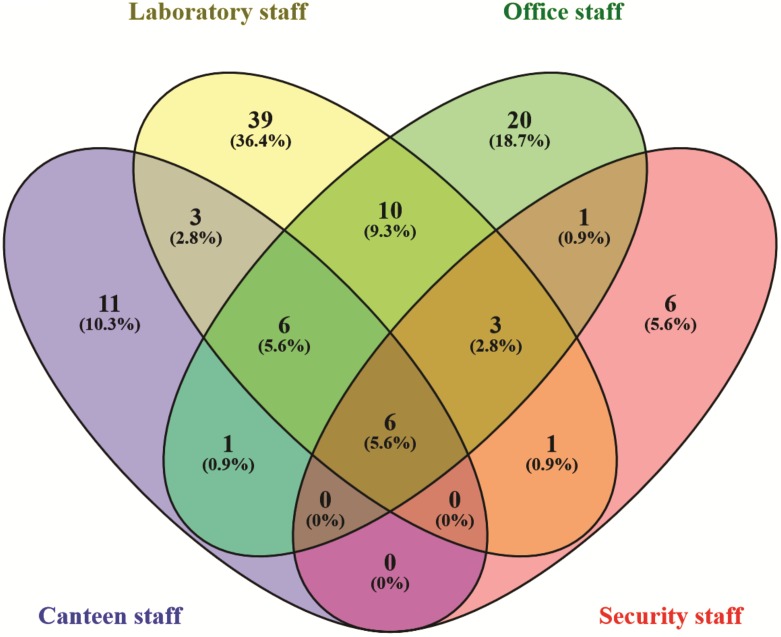
Venn diagram exhibiting the microbial species shared and unique to the individuals working in different settings.

In the Unweighted Pair Group Method with Arithmetic Mean (UPGMA) based dendrogram analysis resulted in grouping of cellular phone samples under three major clades and one independent sample (**Figure 3**). However, this grouping was not specific to the working environment of the cellular phone users, and samples from the different working environment were clustered together to form one group. Principal coordinate analysis (PCoA) biplot based on top three coordinates explained 36.8% variation and clustered the sample in different coordinates (**Figure 4**). All the samples from the cellular phones of canteen staff were placed on the positive side of PCoA1, while all the samples from security staff were placed on the negative side of PCoA2. Majority of the samples from laboratory staff and office staff were placed on the negative side of PCoA1, leaving few expectations on the positive side of PCoA1. The samples from the cellular phones of specific work groups were scattered on the positive and negative side of PCoA 3. The analysis based on the presence and absence of microbial species among the samples of each study groups revealed the presence of six microbial species (viz. *Bacillus altitudinis, Micrococcus luteus, Pseudomonas stutzeri*, *Staphylococcus arlettae, S*. *saprophyticus*, and *S*. *gallinarum*) in all study groups (**Figure 5** and **Supplementary File S3**). In addition to this, six microbial species were common to the cellular phones of office, laboratory, and canteen staff, and three species were shared by the cellular phones of office, laboratory, and security staff. *Aspergillus sydowii, A*. *welwitschiae, Bacillus oceanisediminis, Brevundimonas vesicularis, Candida parapsilosis, C*. *tropicalis, Cellulosimicrobium cellulans, Daldinia starbaeckii, Montagnula scabiosae, Mucor circinelloides*, and *Rhizomucor variabilis* were unique to the cellular phones of canteen staff. Similarly, 39 microbial species were unique to the cellular phones of laboratory staff, 20 microbial species were unique to cellular phones of office staff, and six microbial species including *Brevibacterium epidermidis, Enterobacter cloacae, Exiguobacterium acetylicum, Sphingobium chlorophenolicum, Staphylococcus nepalensis*, and *S*. *xylosus* were unique in the cellular phones of security staff.

### Influence of MALDI-TOF MS Spectrum on Microbial Identification

During this study, we encountered several examples of MALDI-TOF MS spectrum quality influencing the results of microbial identification. The total number of clusters obtained by comparing the MALDI-TOF MS profiles of 112 led to the grouping of these isolates under 39 clusters, and further sequencing based identification of these isolates confirmed the belongingness of these isolates to 30 different microbial species (**Supplementary File S1**). Isolates belonging to microbial species, *Aureobasidium melanogenum*, *Bacillus altitudinis*, *Bacillus subtilis*, *Exiguobacterium acetylicum*, *Pantoea septica*, and *Staphylococcus gallinarum* were represented by more than one MALDI-TOF MS dendrogram group. In addition to this, MALDI-TOF MS biotyper identified few isolates up to species-level, while many others remained unidentified, which were later identified as the member of same species based on sequencing (**Supplementary File S1**). A comparison of MALDI-TOF MS spectra of the isolates belonging to same species revealed that isolates with no reliable identity have the fewer number of mass values in comparison to isolates identified by biotyper database (**Supplementary Figure S1**). In a curious case of isolate K1S22 a poor quality spectrum showed 1.77 score value to *Filifactor villosus* in the biotyper database search, but no reliable identification when a good quality spectrum was generated for the same isolate (**Supplementary Figure S2**).

## Discussion

Exposure of human to microorganisms is inevitable, as trillions of microbial cells surround us. The increasing number of infectious diseases, antibiotic resistance, and numbers of immune-compromised individuals is posing a challenge to devise new methods to minimize microbial exposure. Better sanitisation procedures have been considered a best preventive measure to minimize the microbial exposure leading to the reduced use of antibiotic, hence reducing ‘silent’ spread of antibiotic-resistant strains (Bloomfield et al., 2016). Highly effective sanitisation methods are becoming quite common in the developed world and most of developing countries. Cellular phones are one of the most necessary electronic gadgets across the world and are continuously exposed to diverse environments and in the absence of proper sanitization procedures, cellular phones carry a huge diversity of microorganisms (Meadow et al., 2014). In this study, we isolated a total of 554 isolates from 27 cellular phones, which belong to 107 different microbial species, indicating the diversity of microorganisms residing on the screens of cellular phones (**Figure 1** and **Supplementary File S2**). The number of isolates picked was adequate to express the diversity of cultivable microorganisms, as the number of morphotypes from each sample was high in comparison to the number of species identified from the respective cellular phone (**Figure 2**). Bacteria appear more dominant in comparison to fungi on the cellular phones as the total identified species 71% belonged to 31 different genera of bacteria and 29% belonged to 20 genera of fungi (**Figure 1** and **Supplementary File S2**). During the evaluation of bacterial and fungal contamination in the healthcare workers’ hands and rings in the intensive care unit, showed that most of the samples were colonized with bacteria including 23% staphylococci, 7.9% *Klebsiella* spp., 4.7% *Enterobacter* spp., 3.9% *Escherichia coli*, 3.1% *Acinetobacter* spp., 2.3% *Pseudomonas* spp., and fewer (27.7%) with fungi (Khodavaisy et al., 2011).

Most of the previous studies on cellular phone related microorganisms were specifically focused on the pathogenic microorganisms from hospital settings (Goldblatt et al., 2007; Brady et al., 2011; Lee et al., 2013; Nwankwo et al., 2014; Heyba et al., 2015; Ulger et al., 2015). Majority of cellular phones examined during these studies showed the presence of pathogenic microorganisms, indicating that cellular phones might serve as vectors for transmission infection to patients. In the present study, members of *Staphylococcus*, *Bacillus*, *Micrococcus*, and *Pseudomonas* were the most frequently encountered bacteria, and *Candida*, *Aspergillus*, *Aureobasidium*, and *Cryptococcus* were in case of fungi (**Figure 1A**), members of these genera have been reported frequently from cellular phones surfaces (Khodavaisy et al., 2011; Heyba et al., 2015; Furuhata et al., 2016; Kõljalg et al., 2017). Several members of these genera are opportunistic pathogens and have the ability to colonize the skin and mucous membranes of humans (Brakhage, 2005; Otto, 2009; Chan et al., 2011; de Bentzmann and Plésiat, 2011; Vázquez-González et al., 2013; Kao et al., 2014; Smith et al., 2017; Strauß et al., 2017). Species-level identification of microbial isolates from the cellular phones indicated the prevalence of *Micrococcus luteus*, *Staphylococcus hominis*, *Staphylococcus epidermidis*, *Staphylococcus arlettae*, *Bacillus subtilis*, and *Candida parapsilosis*, which are mostly commensal inhabitants of healthy human skin (**Figure 1B**). *Staphylococcus epidermidis* and other coagulase-negative staphylococci along with coryneforms of the phylum *Actinobacteria* and the genus *Micrococcus* have been regarded as the primary bacterial colonizers of the skin (Grice and Segre, 2011). *Bacillus subtilis* is a ubiquitous organism, and found on the skin, in epithelial wounds, on extremities of the human body, in livestock, and in soil (Earl et al., 2008). Strains of *Bacillus subtilis* have developed various strategies like the secretion of a large number of molecules that control the growth of neighboring organisms and formation of resistant spores to survive in diverse environments. *Candida parapsilosis* is typically a commensal of human skin, but has the capacity to form biofilms on implanted devices, to grow in total parenteral nutrition and for nosocomial spread by hand carriage (Clark et al., 2004; Trofa et al., 2008; Jarros et al., 2018).

The microbial communities associated with all cellular phones are unique as depicted by the UPGMA dendrogram and PCoA biplot (**Figures 3**, **4**). The grouping of all cellular phones belonging to canteen staff on the positive side of coordinate 1, indicated some level of similarity in the microbial communities among these samples (**Figure 4**). Similarly, three samples belonging to security staff were also placed together on the negative side of coordinate 2, showed the selection of specific microbial communities in the cellular phones of individuals working in the similar environments. However, we could not find any specific grouping of individuals working in laboratory and office settings, which might be because these individuals are having some overlaps in their working environments. The majority of microbial strains isolated from the cellular phones might be the transient population, recently a biofilm forming bacterium (*Microbacterium telephonicum*) was also discovered during this study (Rahi et al., 2018). It is expected that the daily upkeep of the cellular phone by the individual will play an important role in the build-up of microbial communities on the cellular phones. Six species were present in the cellular phones of individuals working all four environments, indicating their wide prevalence on cellular phones (**Figure 5** and **Supplementary File S3**). Except for *Pseudomonas stutzeri*, all of these species belong to *Bacillus*, *Micrococcus*, and *Staphylococcus* genera of Gram-positive bacteria, which are member commensal microorganisms of human skin (Grice and Segre, 2011). In addition to these common species, all groups have unique microbial species ranging from 6 to 39, indicating the selection of specific microbial communities in a particular environment (**Figure 5** and **Supplementary File S3**). The unique microbial species isolated from the cellular phones of individuals working in canteen include a high number of fungi, including *Aspergillus sydowii, A*. *welwitschiae, Candida parapsilosis*, *C*. *tropicalis, Daldinia starbaeckii, Montagnula scabiosae, Mucor circinelloides*, and *Rhizomucor variabilis*. Although in this study we have not considered other parameters like personal hygiene and sanitation to correlate with the microbial diversity, and strongly believe that personal hygiene and sanitation measures such as hand washing before handling cellular phones will greatly influence the microbial build-up on cellular phone surfaces. Based on our results, we also suggest adopting efficient and suitable decontamination procedures to lessen the chances of cross contamination via cellular phones. It is obvious that the sample size in this study was modest and it also lacks equal representation of individuals from each working environment, as most of the samples were collected are from individuals working in the laboratory. A wider sampling including the higher number individuals from diverse social categories (i.e., demographic), and use of multiple growth media and conditions will allow us to determine more specific correlations between the presence of objectionable microbial communities and working environments of cellular phone users.

Use of MALDI-TOF MS for microbial identification appeared promising as out of 554 isolates we could identify nearly 80% isolates. In addition to the identification, clustering of unidentified isolates based on MALDI-TOF MS profiles allowed the de-replication of similar isolates, hence reducing the number of isolates from 112 to 39 for further sequence based identification (**Supplementary File S1**). De-replication of microbial isolates based on MALDI-TOF MS is quite fast in comparison to that of based on ERIC-PCR and 16S rRNA gene PCR-RFLP, which involves multiple steps like, genomic DNA isolation, PCR amplification, and gel electrophoresis (Rahi et al., 2012). However, there were few multiple clusters comprising isolates belonging to a single species (**Supplementary File S1**). Inconsistency in the MALDI-TOF MS based identification was revealed by comparing the results of MALDI-TOF MS based identification to that of sequencing as few isolates belonging to *Bacillus subtilis*, *Pseudomonas oryzihabitans*, *Staphylococcus arlettae*, *S*. *haemolyticus*, *S*. *hominis*, and *S*. *warneri*, were not identified by MALDI-TOF MS based identification, while several other isolates belonging to these species were identified MALDI-TOF MS (**Supplementary File S1**). Low quality MALDI-TOF MS spectra (i.e., low peak number) seem responsible for no reliable identification of such isolates by MALDI-TOF MS biotyping (**Supplementary Figure S1**). The quality spectra with a maximum of 100 peek were considered for MALDI-TOF MS based identification (Lagier et al., 2016). Rahi et al. (2016) discussed the role of growth phase in generating the protein spectra, as the extended incubation period might lead to the formation of resistant structures. In this study, it was observed that a poor quality spectrum can also contribute a wrong identification (**Supplementary Figure S2**), where a spectrum of isolate K1S22 belonging to *Exiguobacterium acetylicum* (identified based on 16S rRNA gene sequencing) showed 1.8 score value to *Filifactor villosus*. These observations directed us to conclude that quality of spectrum should be confirmed before making the comparison to the database. Except these few cases, MALDI-TOF MS based identification allowed us to identify this large collection of microorganisms and could not identify in the absence of the spectral database, which is a major challenge for MALDI-TOF MS based identification (Rahi et al., 2016). MALDI-TOF MS is also useful in the discovery of novel species, as in this study three new species including the fungus *Pyrenochaeta telephoni*, and two bacteria *Lysinibacillus telephonicus* and *Microbacterium telephonicum* were described (Crous et al., 2015; Rahi et al., 2017, 2018). It is expected that by performing some basic quality filtering of MALDI-TOF MS spectrum and improving the spectral database will improve the role of MALDI-TOF MS in microbial identification.

## Author Contributions

RK collected samples and isolated and identified microorganisms. DC performed the data analysis and drafted a part of the manuscript. AP performed MALDI-TOF MS based identifications. MK did sequencing based identifications. YS contributed overall supervision and guidance. PR designed the study, analyzed the data, and wrote the manuscript.

## Conflict of Interest Statement

The authors declare that the research was conducted in the absence of any commercial or financial relationships that could be construed as a potential conflict of interest.

## Supplementary Material

FIGURE S1 Matrix-assisted laser desorption ionization time of flight (MALDI-TOF) MS spectra of two isolates indicating the differences in the spectral quality (isolates S5H238 identified as *Bacillus subtilis* by MALDI biotyper database search, whereas not reliable identification was obtained for S2S85), both identified as member of *Bacillus subtilis complex* by 16S rRNA gene sequencing.

FIGURE S2 Matrix-assisted laser desorption ionization time of flight MS spectra of isolate K1S22 indicating the differences in the spectral quality, MALDI biotyper database search for first spectrum **(a)** resulted in genus level identity, whereas not reliable identification was obtained for second spectrum **(b)**, the isolate was identified as *Exiguobacterium acetylicum* by 16S rRNA gene sequencing.

FILE S1 List of isolates identified by 16S rRNA gene and ITS regions sequencing, and their sequence accession numbers.

FILE S2 List of total microbial genera and species identified during the study.

FILE S3 List of microbial species unique and shared by individuals working in different settings.
