# Supplementary material for: Cultivable Microbial Diversity Associated With Cellular Phones
Source: Front Microbiol. 2018 Jun 7;9:1229. doi: 10.3389/fmicb.2018.01229 (PMC6000418; doi:10.3389/fmicb.2018.01229)
Supplement: FIGURE S1 — Matrix-assisted laser desorption ionization time of flight (MALDI-TOF) MS spectra of two isolates indicating the differences in the spectral quality (isolates S5H238 identified as Bacillus subtilis by MALDI biotyper database search, whereas not reliable identification was obtained for S2S85), both identified as member of Bacillus subtilis complex by 16S rRNA gene sequencing. [file Image_1.pdf]

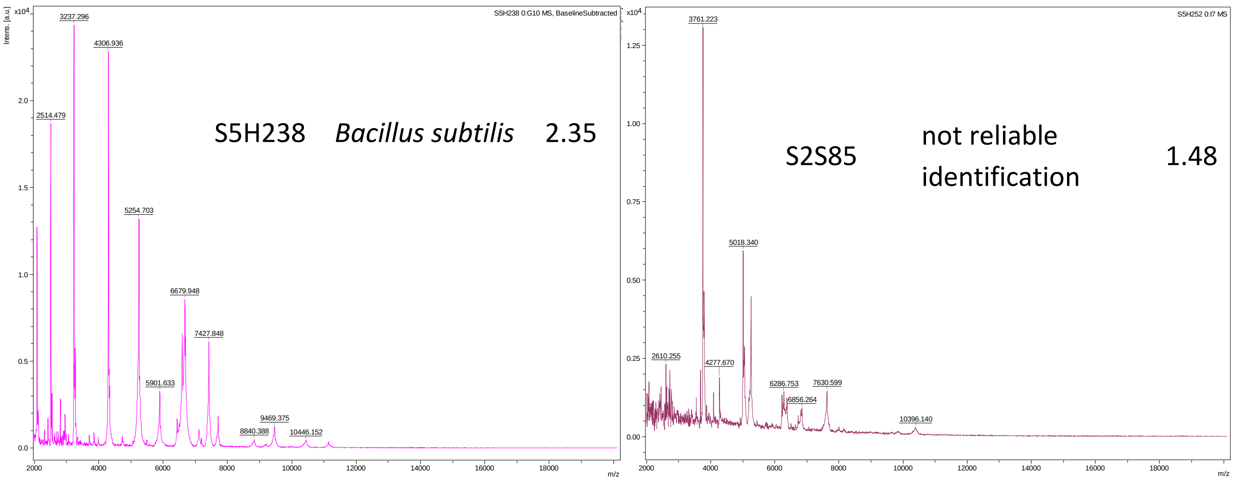

Fig. S1 MALDI-TOF MS spectra of two isolates indicating the differences in the spectral quality (isolates S5H238 identified as *Bacillus subtilis* by MALDI biotyper database search, whereas not reliable identification was obtained for S2S85), both identified as member of *Bacillus subtilis complex* by 16S rRNA gene sequencing.
