# Supplementary material for: Cultivable Microbial Diversity Associated With Cellular Phones
Source: Front Microbiol. 2018 Jun 7;9:1229. doi: 10.3389/fmicb.2018.01229 (PMC6000418; doi:10.3389/fmicb.2018.01229)
Supplement: FIGURE S2 — Matrix-assisted laser desorption ionization time of flight MS spectra of isolate K1S22 indicating the differences in the spectral quality, MALDI biotyper database search for first spectrum (a) resulted in genus level identity, whereas not reliable identification was obtained for second spectrum (b), the isolate was identified as Exiguobacterium acetylicum by 16S rRNA gene sequencing. [file Image_2.pdf]

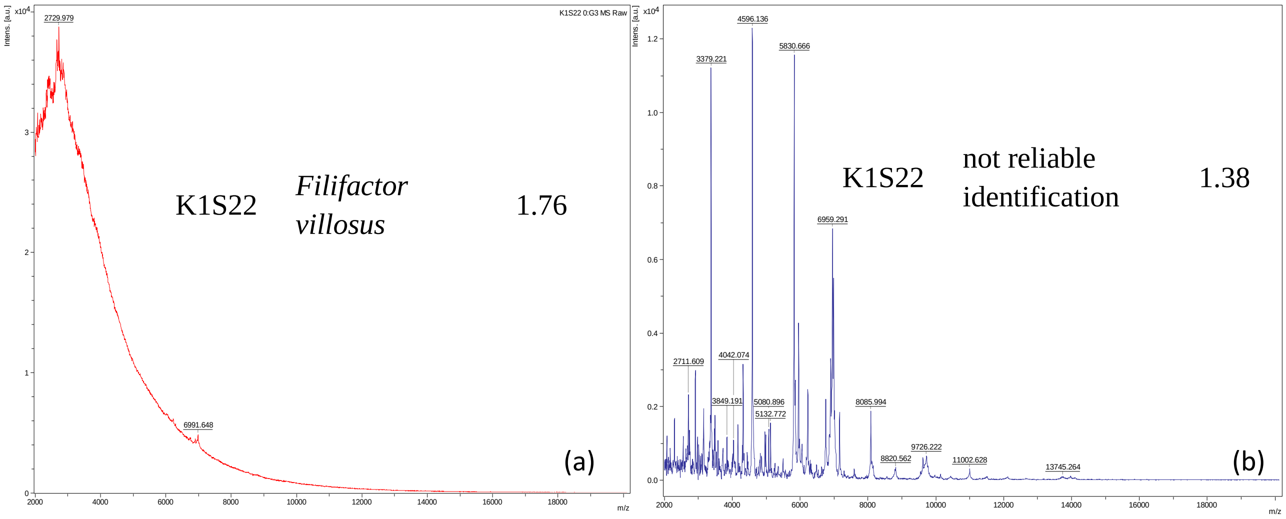

Fig. S2 MALDI-TOF MS spectra of isolate K1S22 indicating the differences in the spectral quality, MALDI biotyper database search for first spectrum (a) resulted genus level identity, whereas not reliable identification was obtained for second spectrum (b), the isolate was identified as *Exiguobacterium acetylicum* by 16S rRNA gene sequencing.
